# A sampling approach to Debiasing the offline evaluation of recommender systems

**DOI:** 10.1007/s10844-021-00651-y

**Published:** 2021-07-10

**Authors:** Diego Carraro, Derek Bridge

**Affiliations:** Insight Centre for Data Analytics, School of Computer Science, Information Technology, University College Cork, Cork, Ireland

**Keywords:** Recommender systems, Offline evaluation, Bias problem, Intervened test sets

## Abstract

Offline evaluation of recommender systems (RSs) mostly relies on historical data, which is often biased. The bias is a result of many confounders that affect the data collection process. In such biased data, user-item interactions are Missing Not At Random (MNAR). Measures of recommender system performance on MNAR test data are unlikely to be reliable indicators of real-world performance unless something is done to mitigate the bias. One widespread way that researchers try to obtain less biased offline evaluation is by designing new, supposedly unbiased performance metrics for use on MNAR test data. We investigate an alternative solution, a *sampling approach*. The general idea is to use a sampling strategy on MNAR data to generate an *intervened* test set with less bias — one in which interactions are Missing At Random (MAR) or, at least, one that is more MAR-like. An existing example of this approach is SKEW, a sampling strategy that aims to adjust for the confounding effect that an item’s popularity has on its likelihood of being observed. In this paper, after extensively surveying the literature on the bias problem in the offline evaluation of RSs, we propose and formulate a novel sampling approach, which we call WTD; we also propose a more practical variant, which we call WTD_H. We compare our methods to SKEW and to two baselines which perform a random intervention on MNAR data. We empirically validate for the first time the effectiveness of SKEW and we show our approach to be a better estimator of the performance that one would obtain on (unbiased) MAR test data. Our strategy benefits from high generality (e.g. it can also be employed for training a recommender) and low overheads (e.g. it does not require any learning).

## Introduction

Offline evaluation of a recommender system is done using an *observed dataset*, which records interactions (e.g. clicks, purchases, ratings) that occur between users and items during a given period in the operation of the recommender system. However, this dataset is *biased*, not only due to the freedom that users have in choosing which items to interact with, but also due to other factors, known as confounders (Chaney et al., 2018; Wang et al., 2018). For example, the recommender system’s user interface acts as a source of confounding factors: differences in the ways that items are exposed to users (e.g. position on the screen) influence the likelihood of a user interacting with those items (Liang et al., 2016b). The recommender itself sets up a feedback loop, which results in another confounder: users are often more likely to interact with the recommender’s suggestions than with other items. The user’s preferences are also a confounder: for example, Marlin et al. demonstrate that, in a dataset of numeric ratings, the probability of not observing a specific user-item interaction depends on the rating value — informally, users tend to rate items that they like (Marlin et al., 2007). Because of these and other confounders, interactions that are missing from an observed dataset are Missing Not At Random (MNAR) (Marlin et al., 2007).

Classical offline evaluations using such an observed dataset are in effect making the assumption that interactions that are missing from the observed dataset are either Missing Completely At Random (MCAR) or Missing At Random (MAR) (Marlin et al., 2007). (For the distinction between MCAR and MAR, see Section 2.2). Using MNAR data in an evaluation as if it were MCAR or MAR, results in biased estimates of a recommender’s performance (Marlin et al., 2007): for example, such experiments tend to incorrectly reward recommenders that recommend popular items or that make recommendations to the more active users (Pradel et al., 2012; Cremonesi et al., 2010).

There are three ways of addressing this problem. The most straightforward approach (in theory, at least) is to collect and employ a MAR dataset, instead of an MNAR one, for the offline evaluation. Using (unbiased) MAR data for the evaluation would give an unbiased estimate of the recommender’s performance. In some domains, there are ways of collecting small MAR-like datasets (see Section 2.3). But, in many domains, it is either impractical or too expensive to obtain MAR-like datasets.

Because of the difficulty of collecting MAR-like data, the other two ways of addressing the problem focus on using MNAR data (which is usually available and in larger quantities) but mitigating its bias. One way of doing this is to design evaluation metrics which compensate for the bias in the MNAR test data. Although this achieves the desired goal to some extent, unbiased metrics suffer from two potential drawbacks. The first is that they may not be general enough to overcome all sources of bias, i.e. they are often designed to compensate for a specific kind of bias: for example, the accuracy metric that is proposed in Steck (2011) is able to correct only for the long-tail popularity bias in a dataset. The second drawback is that the unbiasedness of these metrics might be proven only if the data satisfies some specific conditions: the ATOP estimator proposed in Steck (2010), for example, is unbiased only if the data satisfies certain conditions.

The third approach is to *intervene* on MNAR test data before using it for the evaluation. In practice, such intervention is performed by means of a *sampling strategy* which samples from the available MNAR test data. The sampling strategy is chosen so that the intervened test set which results from the sampling is supposed to be less biased (more MAR-like) and therefore more suitable for evaluation of the recommender’s performance. One such sampling strategy is known as SKEW (Liang et al., 2016a): it samples user-item interactions in inverse proportion to item popularity, thus producing test data with reduced popularity bias.

In this paper, we investigate a new alternative for generating intervened data. Our main contributions are as follows:

- We survey the bias problem in the offline evaluation of RSs and the solutions that have been explored to cope with it. In particular, we describe the use of unbiased datasets and unbiased metrics, as well as the generation of intervened datasets to debias the evaluation; and we discuss the pros and the cons for each of them.
- We propose our own solution to debias the evaluation of an RS. Our method, which we designate WTD (and its variant WTD_H), intervenes on biased data to generate data that is less biased. WTD and WTD_H use a *weighted* sampling strategy in which the weights are calculated by considering the divergence between the distribution of users and items in the MNAR data and their corresponding target (unbiased) MAR distributions.
- We compare WTD and WTD_H with SKEW (Liang et al., 2016a), which is the closest intervention approach to ours. For the first time in the literature, we provide empirical evidence that WTD, WTD_H and SKEW are valid methods to perform the desired debiasing action. With experimental results for two publicly-available datasets, we demonstrate that our solution more closely approximates the unbiased performances of different recommender algorithms. Additionally, we show that it enjoys low overheads and high generality. For example, although in this paper we employ our technique to generate a *test set* for an offline recommender evaluation, our approach is general and can also be employed to debias the data used for training a recommender.

This paper is an extension of Carraro and Bridge (2020), drawing additional material from Carraro (2021). The rest of its content is organised as follows. Section 2 presents related work in the literature. In Section 3, we propose our own contribution to debiasing, which we call WTD (and its variant WTD_H). Section 4 describes the experiments we have run to assess the effectiveness of our approach. We analyse the results of the experiments in Section 5. We discuss our findings and future research directions in Section 6.

## Related work

In this paper, we focus on the offline evaluation of recommender systems. In Section 2.1, we describe a general framework for the classic evaluation of an RS. In Section 2.2, we give an overview of the bias problem that affects such evaluation. Then, in Sections 2.3, 2.4, 2.5, we review three different solutions to this problem that have been explored in the literature.

### Offline evaluation of recommender systems

We consider a recommendation scenario where we define a user-item space, *U* × *I*, of size |*U*|⋅|*I*|. We denote with *u* ∈ *U* = {1,..,|*U*|} a generic user, and with *i* ∈ *I* = {1,..,|*I*|} a generic item. Offline evaluation of a recommender system is done using an *observed dataset* *D*, which, within the user-item space, records interactions that occur between users and items during a given period in the operation of the recommender system platform. Without loss of generality and because our experiments are performed with datasets of explicit ratings (see Section 4), from now on, we will consider such interactions to be numeric ratings.

We visualize *D* as a |*U*|×|*I*| matrix, i.e. HCode $D \in (\mathbb {R} \cup \{\bot \})^{|U| \times |I|}$, where the *r*_*u*,*i*_ entry records the rating given by the user *u* to the item *i* if the rating is observed, ⊥ otherwise. We write *r*_*u*,*i*_ ∈ *D* if the rating is observed, i.e. *r*_*u*,*i*_≠⊥, and *r*_*u*,*i*_∉*D* if the rating is not observed, i.e. *r*_*u*,*i*_ = ⊥. For RS, *D* is typically *sparse*, i.e. the number of observed ratings in *D* is much smaller than all the possible ratings in the whole user-item space. We will write |*D*| for the number of real-valued entries, i.e. |*D*| = |{*r*_*u*,*i*_ ∈ *D*}|. Then, sparsity of the observed dataset means that |*D*| << |*U*|⋅|*I*|. We denote with *D*_*u*_ the observed ratings of the user *u* and with *D*_*i*_ the observed ratings of the item *i*. We will also define the binary random variable ${\mathscr{O}}: U \times I \rightarrow \{0,1\}$ over the set of user-item pairs in *D* as ${\mathscr{O}} = 1$ if the user-item rating is observed (real-valued) and ${\mathscr{O}} = 0$ otherwise (equal to ⊥). (Later, however, when writing probabilities, we will use abbreviation $P({\mathscr{O}})$ in place of $P({\mathscr{O}} = 1)$).

To evaluate an RS, the observed ratings in *D* are typically partitioned into a training set matrix *D*^*tr*^ and a test set matrix *D*^*te*^; more generally, a training set and a test set are sampled from *D*. The training and test sets are typically obtained by performing a random split of the real-valued ratings in *D* but ignoring the values of the ratings. Other, more specific protocols are sometimes used. For example, we might split the ratings on a per-user basis, or we might leverage side information such as the timestamp of the ratings to obtain a temporal split (Koren, 2009; Lathia, 2010).

Once the data is prepared, the experiment consists of using the algorithm under evaluation to train a recommender model on the training set *D*^*tr*^; then, the model is tested using the test set *D*^*te*^ to provide one or more measures of the quality of the algorithm under evaluation. Sometimes this process is performed *k* times, with *k* different training-test splits and results averaged across the *k* experiments.

In early work in this field, there was a focus on accurate prediction of users’ ratings. Hence, experiments used evaluation metrics that compare predicted and actual ratings for items in the test set *D*^*te*^. Examples of such metrics are the Root Mean Square Error (RMSE) and the Mean Absolute Error (MAE). More recently, the focus has shifted to top-*n* recommendation, i.e. whether a recommender model correctly ranks the set of a user’s heldout test items and especially whether it correctly identifies and ranks the first *n* such items. For this, we use evaluation metrics such as Precision, Mean Average Precision (MAP), Recall and Normalized Discounted Cumulative Gain (NDCG). They require a definition of what it means for a test item that is recommended during the experiment to be *relevant*. The typical definition is that a test item *i* is relevant to user *u* if *r*_*u*,*i*_ exceeds some threshold. Specifically, for 1-5 star ratings datasets used in this paper, we define the item *i* to be relevant to the user *u* if *r*_*u*,*i*_ > 3.

### The bias problem

**Fig. 1:**
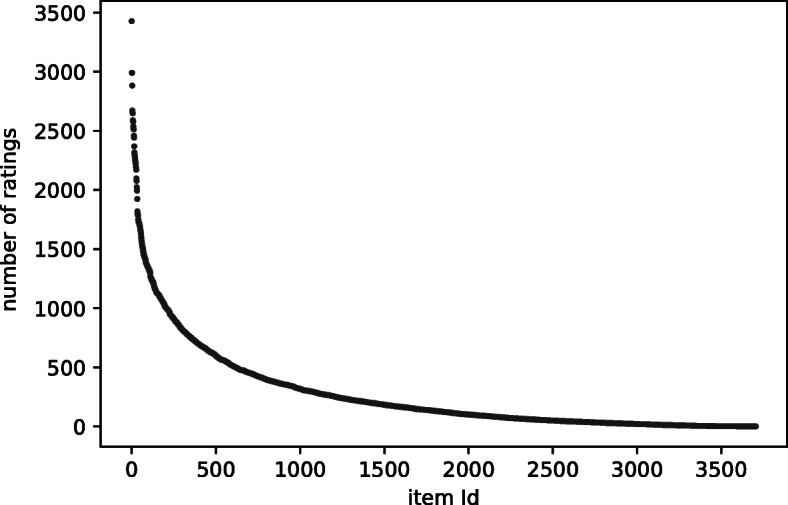
Long-tail popularity curve for Movielens 1M dataset. The items on the *x*-axis are ordered by decreasing number of ratings

It has been widely recognised in the literature that the observed datasets used in the offline evaluation of RSs are *biased*. The bias in a dataset is caused by many factors, known as confounders, that influenced the collection of the dataset (Chaney et al., 2018; Wang et al., 2018). For example, users usually experience what we can call *item discovery bias* because the RS acts as a confounder in the way that items are exposed to users (Cañamares and Castells, 2018). Indeed, the recommender’s user-interface plays an important role as a confounder, e.g. the position of items on the screen influences the likelihood of a user interacting with those items (Liang et al., 2016b). Also, the recommender’s algorithm sets up a feedback loop, which results in another confounder: users are typically more likely to interact with the recommender’s suggestions than with other items. The user’s preferences are also a confounder because they influence whether to consume an item or not (*item consumption bias*) and whether to rate an item or not (*rating decision bias*). In a typical RS scenario, users are free to consume the item if they wish and, usually afterwards, they are free to rate the item or not. Their behaviour is often guided by their preferences on those items: for example, Marlin et al. demonstrate that, in a dataset of numeric ratings, the probability of not observing a specific user-item rating depends on the value associated with that particular rating — informally, users tend to rate items that they like (Marlin et al., 2007). User preferences and the characteristics of an RS are confounders that may also contribute to the so-called *item popularity bias*, i.e. the tendency of users to interact with popular or mainstream items rather than unpopular or niche items. This bias gives rise to the long-tail popularity curve (Hart, 2007), a well-known phenomenon in many RS datasets, where the distribution of the user interactions with items is skewed towards a few popular items (Celma, 2008; Abdollahpouri et al., 2017); see Fig. 1 for an example. There are many publications that measure and explore popularity bias, for example, Pradel et al. (2012) and Abdollahpouri et al. (2017).

Because of these and other confounders, classical offline evaluations, which use biased observed datasets, result in biased (i.e. incorrect) estimates of a recommender’s performance (Marlin et al., 2007). They are *biased evaluations*. For example, such experiments tend to incorrectly reward recommenders that recommend popular items or that make recommendations to the more active users; or they favour recommender approaches that exploit the bias in the dataset (Pradel et al., 2012; Cremonesi et al., 2010; Bellogín et al., 2017).

Work in the RS field that seeks to handle the evaluation bias problem often leverages concepts from the fields of missing data analysis or causal inference. The missing data analysis theories, firstly proposed by Little and Rubin (1986) and later introduced into the recommender systems literature by Marlin et al. (2007), categorise different types of datasets based on so-called *missing data mechanisms*, which describe the process that generates the interaction patterns in the data. According to those theories, interactions that are missing from an observed dataset are Missing Not At Random (MNAR) (Marlin et al., 2007) because of the many confounders, i.e. the dataset is biased. Nevertheless, classical offline evaluations using such an observed dataset are in effect making the assumption that missing interactions are either Missing Completely At Random (MCAR) or Missing At Random (MAR) instead (Marlin et al., 2007). (For the distinction between MCAR and MAR, see below). Using MNAR data in an evaluation as if it were MCAR or MAR results in a biased evaluation.

In work on causal inference, the same missing data mechanisms are typically called the *assignment mechanisms* (Imbens and Rubin, 2015). Roughly speaking, in a recommendation scenario the assignment mechanism exposes users to items and influences the interaction patterns of such users, e.g. analogously to exposing a patient to treatment and later observing its outcome in a medical study. As with the missing data mechanisms, ignoring the biased nature of the assignment mechanism most likely results in a biased evaluation.

In this paper, we use the missing data analysis terminology, i.e. MNAR, MAR, MCAR, and we want to make clearer how we use it in the following. In the literature, a distinction is sometimes drawn between Missing Completely At Random (MCAR) and Missing At Random (MAR). In Little and Rubin (1986) and Marlin et al. (2007), MCAR means that whether a user-item interaction is missing or not does not depend at all on interaction values (such as the values of the ratings in a recommender), i.e. it depends neither on the observed interaction values nor the missing interaction values. MAR, on the other hand, means that whether a user-item interaction is missing or not may depend on the observed interaction values, but is independent of the missing interaction values. However, in this paper, we use MNAR and MAR in a more informal and general way. We use MNAR to indicate that data is biased because missing interactions depend on some confounders. Thus we use the terms MNAR and “biased” interchangeably. We use MAR to refer to data that is unbiased, where missing interactions do not depend on any confounder. Thus we use the terms MAR and “unbiased” interchangeably. Although these more informal usages are not properly in line with the categorisation in Little and Rubin (1986) and Marlin et al. (2007), our choice is broadly in line with other work in the recommender systems literature: what we refer to as MAR is also called MAR in papers such as Steck (2010) and Cañamares and Castells (2018). But what we call MAR is also referred to as MCAR in papers such as Schnabel et al. (2016) and Kim and Choi (2014).

There are three main ways by which experiment designers address the bias problem in the offline evaluation of RSs, and each of them will be reviewed in the following three sections. In Section 2.3, we describe a protocol to collect a MAR-like dataset which can be used instead of an MNAR dataset for the offline evaluation. In Sections 2.4 and 2.5, we describe unbiased metrics and debiasing interventions, respectively — two solutions that allow ‘debiased’ evaluations on MNAR data. Other approaches to unbiased evaluation have been published in recent years but in the specific context of RS that use Reinforcement Learning. A few examples for the interested reader are Huang et al. (2020), Ie et al. (2019), and Shi et al. (2019) We do not review these in any further detail because they are only partially related to the classical way of evaluating RSs (Section 2.1) and they do not always explicitly address the bias problem, tackling it only indirectly. There is also a substantial body of work that has been done in the last few years to cope with bias during the *training* of RSs models, but we do not review this either, since this paper focuses on the *evaluation* of RSs only.

### Collection of unbiased datasets

The ‘straightforward’ approach for coping with bias in the offline evaluation of an RS is to separately collect some unbiased data and use it as the test set. This can be done with what is sometimes called a “forced ratings approach” Cañamares and Castells (2018). For a ratings dataset, user-item pairs are chosen uniformly at random and for each user-item pair that gets selected the user is required (“forced”) to provide a rating for the item. Thus, randomly-selected users are required to rate randomly-selected items. A dataset that is collected in this way will largely not exhibit the biases that we find in datasets that are collected during the normal operation of an RS (see Section 2.2). For example, we get rid of the item discovery bias and the popularity bias because items are randomly chosen: no confounders play any role in their selection. Items are not ones that are being exposed by the RS on the one hand, and users are not free to select them on the other hand (Cañamares & Castells, 2018); therefore, it is unlikely that we observe long-tail phenomenona in such datasets. The forced ratings approach also removes the item consumption bias because, users are forced to consume or interact with the item so that they can rate it Cañamares and Castells (2018). (Since the user must rate the item, the only legitimate reason for not consuming the item before supplying the rating is if the item was already known to the user). In a typical RS scenario, by contrast, users are free to consume the item if they wish. The rating decision bias is also removed because users are not free to decide whether or not to rate the chosen item; they are required to rate it Cañamares and Castells (2018).

However, datasets collected by the forced ratings approach are MAR-like, rather than MAR: they may still carry some bias. When building such a dataset, for example, although invitations are sent to users who are chosen uniformly at random, those who agree to participate may be atypical, thus introducing bias. Equally, the fact that, for each user, items to rate are presented sequentially introduces bias: the rating a user assigns to a particular item may be influenced by the items she has rated so far. Although this means that these datasets are less biased, rather than unbiased, to the best of our knowledge, this is still the best way of collecting this type of data.

Furthermore, the forced ratings approach can only work in certain domains; for example, it requires that a user who is presented with an item can quickly consume that item (or part of it) in order to form an opinion of it. In the movie domain, for example, we almost certainly cannot require a user to watch an entire movie (although we could require them to watch a movie trailer). Similarly, the forced ratings approach is impracticable in a tourism domain where a recommender suggests point-of-interests to its users: users cannot really be expected to visit the selected places in order to ‘consume’ and rate them (although we could require them to watch an advertisement video about such places).

Datasets collected by the forced ratings approach include Webscope R3 (Marlin et al., 2007) and cm100k (Cañamares & Castells, 2018) in the music domain, and CoatShopping (Schnabel et al., 2016) in the clothing domain. We will present these datasets in more detail and use them in the experiments of Section 4.

### Unbiased metrics

The majority of the literature tries to overcome the bias in an MNAR test set by proposing new evaluation metrics which provide unbiased or nearly unbiased measures of performance on the MNAR test data. Such measures are supposed to be less affected by the bias in the data and, therefore, more suitable for estimating the true performance of a recommender model. In some of the RS literature, e.g. Schnabel et al. (2016), Steck (2010, 2011) and Yang et al. (2018), metrics of this kind are often called ‘estimators’: estimators are used in statistics to calculate an estimate of a given quantity of interest (a recommender’s performance in our case) from observed data (test set data in our case). This terminology might suit an RS evaluation that takes a statistical framework perspective, e.g. Schnabel et al. (2016). However, in this paper, we prefer the term ‘metric’ to indicate a ‘tool’ that measures a recommender performance based on available test data.

In Steck (2010, 2013), Steck designs ATOP, a new ranking metric that corrects for the biased measurements of Recall on an MNAR test set. However, this metric is unbiased only under two assumptions that must hold for the test set data. The first is that relevant ratings (which are typically a tiny fraction of all the possible ratings in the user-item space) are Missing At Random in the observed data. The second, regarding the non-relevant ratings, is that they are missing with a higher probability than the relevant ratings. In practice, the two assumptions allow the author to ‘ignore’ the missing data mechanism for non-relevant missing ratings (i.e. no missing data model is required). Also, there is no need for a missing data model for the missing relevant ratings at all (because they are missing at random). However, unbiasedness of ATOP is not always guaranteed, i.e. in datasets where these assumptions are unrealistic.

There is also work that tries to tackle specific biases in the data. For example, in Steck (2011), Steck designs a modified version of the Recall metric that corrects for the long-tail item popularity bias. He modifies the definition of Recall by introducing weights that are proportional to the inverse popularity of the test set items. The resulting metric, which he calls Popularity-Stratified Recall, is considered a nearly unbiased metric under the assumption that no other confounders besides item popularity bias occur in the test data.

In Schnabel et al. (2016), the authors derive ‘unbiased’ versions of many widely-used metrics, both for ratings prediction (e.g. MAE and Mean Square Error) and top-*n* recommendation (e.g. Precision and nDCG). The ‘unbiased’ versions are based on the concept of Inverse-Propensity-Scoring (IPS) (Imbens and Rubin, 2015; Little & Rubin, 1986; Thompson, 2012). A propensity score of a particular user-item pair *P*_*u*,*i*_ is the probability of that pair being observed. IPS-based metrics use the propensity scores to weight the prediction/ranking errors on the test data computed by one of the standard metrics above. Schnabel et al. propose two different ways of estimating propensities for MNAR ratings datasets: one using Naive Bayes, and the other using Logistic Regression. While the former is an inexpensive approach, it requires a sample of MAR data; their approach to the latter does not require any additional MAR data but it is instead more expensive and requires additional data (side data) about users and items (e.g. user gender and item features).

There is other work that uses IPS-based techniques to design unbiased metrics. For example, similarly to Schnabel et al. (2016), Yang et al. propose new unbiased metrics to obtain widely-employed ranking measures (e.g. Recall and nDCG) on implicit MNAR datasets (2018). The propensity score is modelled around the concept of item popularity and, in practice, it is calculated as the product of the probability that an item is recommended to the user and the probability that the user interacts with the item (given that the item has been recommended). However, the calculation makes strong assumptions about how data is generated. The assumptions include, for example, that the propensity scores are user-independent (i.e. *P*_*u*,*i*_ = *P*_*i*_); that the user interacts with all the items she likes in the recommended set; and that her preferences are not affected by such recommendations. These assumptions do not hold in general, thus limiting the usefulness of this framework.

Lim et al. also propose a metric for implicit MNAR datasets (2015). They first assume a missing data model under which the observed dataset has been collected. Essentially, this model is that items that are relevant to users are Missing At Random. (This is like one of the assumptions in Steck (2010)). Then, they design a novel evaluation measure, which they call Average Discounted Gain (ADG), that is built upon the nDCG metric. Unlike nDCG, they show that ADG allows unbiased estimation of top-*n* recommendation performances on test data which complies with their missing data model.

Finally, Krichene and Rendle (2020) present another interesting piece of work on implicit datasets that investigates the effectiveness of what the authors call ‘sampled metrics’. Sampled metrics are common evaluation metrics such as, for example, Precision and Recall, but used to measure a recommender’s quality with a testing procedure that speeds up the evaluation (i.e. typically, the evaluation is performed by randomly sampling a small set of irrelevant items and ranking the relevant test set items only among this smaller set, instead of ranking test set items against the entire item catalogue), e.g. Cremonesi et al. (2010) and Ebesu et al. (2018). The authors show that a sampled metric can be a poor estimator of the true performances of recommender algorithms and suggest that the use of sampling in the evaluation should be avoided when possible. However, when sampling is required, the authors propose modifications that correct sampled metric measurements and give a better estimate of the true performances (however, at the cost of increased variance in the results).

Although unbiased metrics, to some extent, achieve the desired goal of obtaining ‘unbiased’ measures of a recommender’s performance, they suffer from some potential drawbacks. One of these is that they may not be general enough to overcome all sources of bias, i.e. they are often designed to compensate for a specific kind of bias (e.g. the popularity bias in Steck (2011)). Another is that their unbiasedness might be proven only if the data used satisfies some specific conditions (e.g. the assumptions of Steck (2010, 2011) and Lim et al. (2015) or the somewhat artificial recommendation scenario in Yang et al. (2018)). Another drawback is that unbiased metrics might need additional data (e.g user gender and item features in Schnabel et al. (2016)). Finally, they might require computationally expensive calculations (e.g. to estimate propensities in Schnabel et al. (2016)).

### Intervened datasets

The third solution to the problem of bias uses what we will call an *intervention* approach, in contrast with what we might call the *regular* approach (in which there is no intervention). In the latter, which is widely used in literature and which was explained earlier, the test set is typically generated by randomly sampling a portion of the available MNAR data, which gives rise to a biased RS evaluation. The former, instead, uses non-random sampling to produce a MAR-like test set, the *intervened test set*, which is supposedly less biased. The intervened test set is used in place of the regular (MNAR) test set to perform an unbiased RS evaluation.

The SKEW method by Liang et al. (2016a) samples user-item pairs in inverse proportion to the item’s popularity. This generates an intervened test set which has roughly uniform exposure distribution across items, thus reducing the item popularity bias in the test set. Liang and co-authors in Liang et al. (2016a) and Wang et al. (2018) and Bonner and Vasile (2018) use this technique for test set generation to evaluate causal approaches to recommendation. However, none of the three works that we have just cited either explain or verify empirically why SKEW should be effective as a debiasing technique. In this paper, we fill the gap by providing such contributions (see Section 4). Also, because of the similarity with our own work on debiased RS evaluation, we use SKEW as a state-of-the-art strategy to compare against our own approach (WTD and WTD_H, see Section 3).

Cremonesi et al. construct an intervened test set by removing ratings for the most popular items in the dataset from the MNAR test set, with the goal of mitigating the item popularity bias of the evaluation (2010). In this way, a recommender’s quality is assessed on long-tail items only, while the recommendation of frequently-rated items is ignored. This is different from SKEW, which does not remove popular items but, rather, samples in inverse proportion to item popularity. Discarding all popular items may lead to specific insights but is generally too restrictive for a comprehensive evaluation. There is also a technical difficulty: given a specific dataset, it is not always clear what proportion of the items should be removed, leaving the evaluation quite arbitrary.

Bellogin et al. also sample an MNAR dataset to try to overcome the item popularity bias in the evaluation of a recommender, by means of two approaches (2017). Their first approach (which is a percentile-based approach) is a form of stratification, in which training and test ratings are sampled from a partition of the data. In practice, the set of items is partitioned into *m* bins, based on item popularity, and the ratings of the items belonging to a bin form a popularity stratum. Then, for each stratum: a training set and a test set are sampled (typically by means of a random split of the ratings available in the stratum); and a recommender model is trained on the training set and tested on the test set. Results for the whole evaluation are obtained by averaging the recommender’s performance across the *m* strata. One drawback of this methodology is the need to choose a value for the parameter *m*: it is not clear what *m* should be. The fact that the whole evaluation is broken down into *m* experiments is another drawback. The consequence is that an evaluation of this kind assesses to what extent a recommender is good at recommending items within a given popularity stratum. Bellogin et al.’s second approach (which they call Uniform Test Item Profiles) builds a test set with the same number of ratings for each item. However, this approach is very sensitive to the steepness of the item popularity curve. It may result in: generating quite small tests sets; and generating test sets where only a few popular items are included, therefore limiting the scope of the evaluation.

## Our approach to Debiased offline evaluation of recommender systems

Designing an offline evaluation methodology which overcomes the bias problem in the data is crucial to obtaining reliable estimates of recommender performance. In Section 2.1, we have presented different solutions that can be found in the literature of the field. In this section, we explain and evaluate our own contribution to debiasing, which we call WTD (and its variant WTD_H). WTD and WTD_H are intervention methods (Section 2.5), where the intervention is performed on MNAR data before using it for the evaluation.

In this section, we first analyse properties of MAR and MNAR data (Sections 3.1 and 3.2). Subsequently, we use those properties to shape our WTD/WTD_H intervention, a sampling strategy in which sampling weights are calculated by considering the divergence between the distribution of users and items in the MNAR data and their corresponding target MAR distributions (Section 3.3).

### Properties of a MAR dataset

Using the notation presented in Section 2.1, we refer to two kinds of datasets over the same *U* × *I* space, $D^{mar} \in (\mathbb {R} \cup \{\bot \})^{|U| \times |I|}$ and $D^{mnar} \in (\mathbb {R} \cup \{\bot \})^{|U| \times |I|}$, which have MAR and MNAR properties respectively. In this section, we analyse properties of *D*^*mar*^ and in the next section the ones of *D*^*mnar*^.

To generate *D*^*mar*^, we use the forced ratings approach that we described in Section 2.3. First, we randomly sample a set of user-item pairs. Then, a preference for each pair is collected so that *D*^*mar*^ is obtained. (As before, without loss of generality, we consider such a preference to be a numeric rating). Note that, in order to satisfy the MAR property, the generation of *D*^*mar*^ is totally independent from the interaction values collected and from the particular identity of the user-item pair (*u*,*i*) as well. We also assume that, once the set of user-item pairs is determined, we can obtain the interaction values for all such pairs. (In practice, of course, users may decline the invitation to participate or may refuse to give some ratings, which is one reason why in reality these datasets are MAR-like and not MAR).

Practically, to sample the set of user-items pairs, we make use of the probability distribution $P_{mar}({\mathscr{O}}|u,i)$, defined over the space *U* × *I*, that leads to *D*^*mar*^. (We recall that we use the binary random variable ${\mathscr{O}}$ to indicate whether a rating is observed or not). A straightforward choice is to set $P_{mar}({\mathscr{O}}|u,i) = P({\mathscr{O}}) = \rho _{mar}$, where *ρ*_*mar*_ represents the desired ratio of observed entries from *U* × *I*.

Now, assuming that a dataset *D*^*mar*^ has been collected using such an approach, we should empirically verify that user and item posterior probabilities are (roughly) uniformly distributed:

1 $$  P_{mar}(u|\mathscr{O}) = \frac{|D^{mar}_{u}|}{|D^{mar}|} \approx \frac{1}{|U|} \quad \forall u \in U $$

2 $$  P_{mar}(i|\mathscr{O}) = \frac{|D^{mar}_{i}|}{|D^{mar}|} \approx \frac{1}{|I|} \qquad \forall i \in I $$

where $D^{mar}_{u}$ and $D^{mar}_{i}$ are the observed ratings for user *u* and item *i* respectively.

Also, because users and items are drawn independently, we have that their posteriors are independent and we can write:

3 $$  P_{mar}(u,i|\mathscr{O}) = P_{mar}(u|\mathscr{O}) P_{mar}(i|\mathscr{O}) \approx \frac{1}{|U||I|} \quad \forall (u,i) \in U \times I $$

for the joint posterior of a specific user-item pair.

### Properties of an MNAR dataset

MNAR data is, of course, usually collected during the operation of a recommender platform. But, similarly to the way we modelled the generation of MAR data *D*^*mar*^, we can model the generation of an MNAR dataset *D*^*mnar*^ in terms of a drawing process.

Differently from the MAR scenario, due to the presence of bias, we cannot assume the sampling distribution *P*_*mnar*_ to be independent from the rating values *D*^*mnar*^ (or from other confounders too, including, e.g., the specific user-item pair (*u*,*i*)). In other words, in an MNAR dataset the draw is generally guided by some unknown probability $P_{mnar}({\mathscr{O}}|u,i, Y, \mathcal {X})$, where *Y* represents the complete set of user-item ratings and $\mathcal {X}$ represents a set of features (covariates, confounders) which influences the sampling probability (e.g. user demographics, item features, characteristics of the system such as the way it exposes items to users, and so on).

If an MNAR dataset *D*^*mnar*^ has been collected, we can examine its user and item posterior probabilities, as we did for the MAR dataset but now, in general, we will find:

4 $$  P_{mnar}(u|\mathscr{O}) = \frac{|D^{mnar}_{u}|}{|D^{mnar}|} \neq \frac{1}{|U|} \quad \forall u \in U $$

5 $$  P_{mnar}(i|\mathscr{O}) = \frac{|D^{mnar}_{i}|}{|D^{mnar}|} \neq \frac{1}{|I|} \qquad \forall i \in I $$

In general, the users and items are not uniformly distributed and thus, given that a specific entry is observed, i.e. HCode ${\mathscr{O}} = 1$, we cannot assume user and item posterior independence for the joint posterior $P_{mnar}(u,i|{\mathscr{O}})$, i.e.

6 $$  P_{mnar}(u,i|\mathscr{O}) \neq P_{mnar}(u|\mathscr{O}) P_{mnar}(i|\mathscr{O}) \quad \forall (u,i) \in U \times I $$

However, the formulation that we have given here provides us with a solid framework to design our debiasing strategy in the next section.

### Intervened test sets

To conduct unbiased evaluation from biased data, we generate and use intervened test sets in place of classical random heldout test sets. We begin by presenting this approach in general (Section 3.3.1), and then we present the specifics of our approach (Sections 3.3.2 and 3.3.3).

#### The intervention approach

The intervention approach consists in performing a debiasing intervention on MNAR data *D*^*mnar*^ by means of a given sampling strategy, denoted with *S*. The result of the intervention is the dataset *D*^*S*^ such that |*D*^*S*^|≤|*D*^*mnar*^| and with the objective that *D*^*S*^ has unbiased-like properties. Formally, we denote with ${\mathscr{S}}:U \times I \rightarrow \{0,1\}$ the binary random variable that takes the value 1 when a particular user-item pair is sampled from *D*^*mnar*^, 0 otherwise. (Again, we will use abbreviation $P({\mathscr{S}})$ in place of $P({\mathscr{S}} = 1)$.) A particular strategy *S* is characterized by the expression of the probability $P_{S}({\mathscr{S}}|u,i, {\mathscr{O}}), \forall (u,i) \in D^{mnar}$, which is the probability distribution responsible for guiding the sampling on *D*^*mnar*^. (In practice, only user-item pairs where a real-valued rating is available in *D*^*mnar*^ can be sampled). We present our sampling approach in the next section.

#### WTD: weights for the sampling

In this section we present WTD, our debiasing intervention on MNAR data: the main idea is to make an intervened test *D*^*S*^ set that resembles a MAR test set in terms of its user-item posterior probabilities; these probabilities are typically uniformly distributed in a MAR test set. In other words, we want to make *D*^*S*^ similar to *D*^*mar*^ in terms of its posteriors by means of a sampling strategy that leverages specific user and item weights. The role of the weights is to adjust the probability of sampling a specific user-item pair in the MNAR data in a way that the resulting sampled *D*^*S*^ has posteriors similar to a MAR.

Formally, we will start by assuming the availability of some MAR-like data *D*^*mar*^ in addition to MNAR data *D*^*mnar*^. In fact, we will see in Section 3.3.3 that we can use our approach even in cases where we do not have any MAR data. We want to make the posterior probability distribution of each user-item pair in the sampled *D*^*S*^, i.e. HCode $P_{S}(u,i|{\mathscr{S}})$, approximately the same as the posterior probability distribution observed for the corresponding user-item pair in *D*^*mar*^, i.e. HCode $P_{mar}(u,i|{\mathscr{O}})$. Writing this as a formula, we want:

7 $$  P_{S}(u,i|\mathscr{S}) \approx P_{mar}(u,i|\mathscr{O}) \quad \forall (u,i) \in D^{S} $$

To obtain this approximation, we adjust the posterior distributions of the sampling space *D*^*mnar*^, i.e. HCode $P_{mnar}(u,i|{\mathscr{O}})$, using user-item weights *w* = (*w*_*u*,*i*_)_*u*∈*U*,*i*∈*I*_ (similarly to Myttenaere et al. (2014)). We denote the modified weighted MNAR posteriors by $P_{mnar}(u,i|{\mathscr{O}}, w)$. The goal is to find weights *w* so that:

8 $$  P_{mnar}(u,i|\mathscr{O}, w) = P_{mar}(u,i|\mathscr{O}) \quad \forall (u,i) \in D^{mnar} $$

From the fact that a typical MAR dataset is uniformly distributed over users and items, we use the independence of Formula (3) to re-write the right-hand side of Formula (8) to obtain:

9 $$  P_{mnar}(u,i|\mathscr{O}, w) = P_{mar}(i|\mathscr{O}) P_{mar}(u|\mathscr{O}) \quad \forall (u,i) \in D^{mnar} $$

Similarly to Formula (6), which considers user-item MNAR joint posteriors $P_{mnar}(u,i|{\mathscr{O}})$, also the user-item *weighted* MNAR joint posteriors $P_{mnar}(u,i|{\mathscr{O}}, w)$ will not in general be independent for the same reason. However, we are going to treat them as if they were independent; we justify this by showing empirically in Section 5 that it does obtain good results. We will then write:

10 $$  P_{mnar}(u,i|\mathscr{O}, w) = P_{mnar}(i|\mathscr{O}, w) P_{mnar}(u|\mathscr{O}, w) \quad \forall (u,i) \in D^{mnar} $$

Now, using Formula (10), we can split Formula (9) into the two following equations:

11 $$  P_{mnar}(u|\mathscr{O}, w) = P_{mar}(u|\mathscr{O}) \quad \forall u \in U $$

12 $$  P_{mnar}(i|\mathscr{O}, w) = P_{mar}(i|\mathscr{O}) \quad \forall i \in I $$

As a consequence of Formulas (11) and (12) for the weighted MNAR posteriors, we can define and calculate user-specific weights *w* = (*w*_*u*_)_*u*∈*U*_ and item-specific weights *w* = (*w*_*i*_)_*i*∈*I*_ instead of weights that are user-item specific. Having independent user and item weights also has an advantage in terms of scalability. We need to calculate only |*U*| + |*I*| weights instead of |*U* × *I*|. This is good for scalability because |*U* × *I*| >> |*U*| + |*I*| for the values of |*U*| and |*I*| that we typically find in recommender domains.

We propose the most straightforward solution to model the weighted MNAR posteriors, i.e. HCode $P_{mnar}(.|{\mathscr{O}}, w) = w_. P_{mnar}(.|{\mathscr{O}})$. We plug this into Formulas (11) and (12) and we obtain $ w_{u} P_{mnar}(u|{\mathscr{O}}) = P_{mar}(u|{\mathscr{O}})$, $ w_{i} P_{mnar}(i|{\mathscr{O}}) = P_{mar}(i|{\mathscr{O}})$ for each user and item weighted distribution respectively. Simply reversing these last two formulas, we have the expressions for calculating the weights:

13 $$  w_{u} = \frac{P_{mar}(u|\mathscr{O})}{P_{mnar}(u|\mathscr{O})} \quad \forall u \in U $$

14 $$  w_{i} = \frac{P_{mar}(i|\mathscr{O})}{P_{mnar}(i|\mathscr{O})} \quad \forall i \in I $$

We can think of the calculated weights as quantities that measure the divergence between the MNAR distributions of the sampling space and the target MAR distribution. Because a specific weight adjusts the corresponding MNAR distribution, we directly use weights to model the sampling distribution, i.e. HCode $P_{S}({\mathscr{S}}|u,i) = w_{u} \times w_{i}$. During the sampling, the effect of the weights is to increase or decrease the probability that a particular user-item pair is sampled depending on how divergent are the user and item posterior probabilities in the MNAR sampling space with respect to the MAR distributions.

In preliminary experiments using $P_{S}({\mathscr{S}}|u,i) = w_{u} \times w_{i}$, we found that our intervened test sets only partly resembled MAR test sets. In Section 4, we use $P_{S}({\mathscr{S}}|u,i) = w_{u} \times (w_{i})^{2}$ for our comprehensive experiments instead. This variant, denoted by WTD in the rest of this paper, raises the importance of the item-weight relative to the user weight. Specifically, (*w*_*i*_)^2^ will be bigger than *w*_*i*_ if *w*_*i*_ is greater than one, and (*w*_*i*_)^2^ will be smaller than *w*_*i*_ if *w*_*i*_ is less than one. This choice makes sense in the light of previous research reported in the literature which identifies item popularity as one of the most impactful confounders in MNAR data, e.g. Pradel et al. (2012) and Steck (2011).

#### WTD_H: hypothesized distributions for the weights

Up to this point, we assumed the availability of some MAR-like data in order to give us the posteriors that we need to approximate. But MAR-like data is expensive or impossible to collect, as we discussed when presenting the “forced ratings approach” earlier. Furthermore, in those cases where we do have a reasonable amount of MAR-like data at hand, we could use it directly as an unbiased test set. Using it to calculate weights so that we can intervene on MNAR data to produce a more MAR-like test set would then be pointless.

In fact, when we do not have any MAR-like data, we can still use our approach. We know that the posterior probability distribution for MAR data is uniform ($P_{mar}(u|{\mathscr{O}}) = 1 / |U|$, $P_{mar}(i|{\mathscr{O}}) = 1 / |I|$), and this is all we need for our sampling approach. Therefore, we can use this hypothesized distribution when calculating the weights, avoiding the need for a MAR-like dataset. We call this strategy, WTD_H (where the H stands for “hypothesized”).

## Experiments

The goal of the offline experiments presented in this section is to assess the ‘goodness’ of different ways of producing intervened test sets. The measure of ‘goodness’ is how much results obtained by evaluating a recommender on an intervened test set resemble the results we would obtain on an unbiased test set. We assess our solutions, i.e. WTD and WTD_H, and compare them to SKEW (Liang et al., 2016a) and to two baselines, FULL and REG. We consider SKEW, which we presented in Section 2.5, to be the state-of-the-art strategy that most closely relates to our approach; FULL and REG perform a non intervention and a random intervention (which, in practice, is equivalent to no intervention) on MNAR data, respectively.

When deciding which intervention strategies to include in our investigation, we discarded some of the ones described in Section 2.5. Cremonesi et al.’s approach (2010) is one of them because it generates a test set devoid of ratings on the most popular items: it turns out that, by doing this, it is impossible to assess the quality of a recommender when recommending popular items, thus limiting the evaluation. We also do not include the two strategies of Bellogin et al., i.e. the percentile-based approach and the Uniform Test Item Profiles approach (2017). The percentile-based approach trains a recommender and tests its performance on separate popularity segments of the item catalogue. Even though the quality of a recommender is inferred by averaging the performances across the segments, we argue that this approach still carries a similar limitation to Cremonesi et al.’s one (i.e. it compromises the representativeness of the whole experiment). The Uniform Test Item Profiles method also most likely discards ratings of some items (the least popular ones this time); and it may result in quite small test sets if the long-tail curve is very steep.

### Datasets

We use two publicly available ratings datasets: Webscope R3^1^ (WBR3) from the music domain (Marlin et al., 2007) and CoatShopping^2^ (COAT) from the clothing domain (Schnabel et al., 2016). Both of them are ideal for our purposes because they are composed of two parts, one having MAR properties (*D*^*mar*^), and the other having MNAR properties (*D*^*mnar*^). However, the two datasets have been collected in quite different recommender scenarios which, we argue, might influence our experimental results (see Section 5). Note that we did mention earlier (Section 2.5) that we know of one other MAR-like dataset, collected by the forced ratings approach, namely cm100k from the music domain (Cañamares and Castells, 2018), but we cannot use this in our experiments because it does not have any corresponding MNAR data.

COAT’s users are Amazon Mechanical Turkers who were asked (through a simple web-shop interface with facets and paging) firstly to find the coat they would have liked to buy the most and, afterwards, to freely rate 24 coats among the ones they had explored; those are the ratings that compose the *D*^*mnar*^ portion of the dataset. It is not clear for how long users were allowed to interact with the system. The forced ratings approach described earlier was used to additionally collect the *D*^*mar*^ portion of the dataset.

For WBR3, data was collected over a 20 day window. During this period, users used the LaunchCast Radio player, which gave them the freedom to rate songs (at any time and in any quantity) and receive personalised recommendations, and this produced the *D*^*mnar*^ portion of the dataset. Again, additionally the *D*^*mar*^ portion was collected using the forced ratings approach. It follows, for the reasons we gave earlier (see Section 2.5), that the *D*^*mar*^ portions of both WBR3 and COAT are almost but not completely unbiased.

For both datasets, ratings are on a 1 to 5 scale and we consider an item as relevant to a user if the item has a rating above 3, non-relevant otherwise.

For each dataset, we applied a preprocessing step to ensure that both *D*^*mar*^ and *D*^*mnar*^ have a common user-item space *U* × *I*: specifically, we keep those users and items that belong to the intersection of the two portions. Table 1 gives statistics of the final resulting datasets that we used in the experiments.

**Table 1:** Datasets statistics

WBR3	MAR	$\sim $54k ratings; 5400 users; 1000 items; 0.99 sparsity
		avg 10 ratings per user; avg 54 ratings per item; 1.81 avg rating value
	MNAR	$\sim $129k ratings; 5400 users; 1000 items; 0.97 sparsity
		avg 23 ratings per user; avg 129 ratings per item; 2.87 avg rating value
COAT	MAR	$\sim $4640k ratings; 290 users; 300 items; 0.94 sparsity
		avg 16 ratings per user; avg 15 ratings per item; 2.22 avg rating value
	MNAR	$\sim $6960k ratings; 290 users; 300 items; 0.92 sparsity
		avg 24 ratings per user; avg 23 ratings per item; 2.61 avg rating value

### Methodology

In our experiments, we randomly split *D*^*mnar*^ in each dataset into a training set *D*^*tr*^ and a heldout set *D*^*he*^ with proportions 60%-40% respectively. Since the split is random, MNAR distributions are preserved. *D*^*he*^ is what one would use as a traditional test set. But, in our case, we use *D*^*he*^ as the sampling space: we sample it to obtain different intervened test sets *D*^*S*^. For each sampling strategy (REG, SKEW, WTD, WTD_H, explained in Section 4.3), we generate 10 different intervened test sets, each of which is obtained by sampling a portion *ρ*^*p*^ from *D*^*he*^. The parameter *ρ*^*p*^ takes all the values in {0.1,0.2,..,1} and represents the size of *D*^*S*^ with respect to the size of *D*^*he*^ (e.g. *ρ*^*p*^ = 0.5 means that |*D*^*S*^| = 0.5|*D*^*he*^|). We can view *ρ*^*p*^ as the parameter that guides the strength of the debiasing action on *D*^*he*^: the smaller is *ρ*^*p*^, the smaller but more debiased is *D*^*S*^; the bigger is *ρ*^*p*^, the bigger and less debiased is *D*^*S*^, i.e. because it is more similar to *D*^*he*^. In Section 5, we will see the impact of different *ρ*^*p*^ values on the results. Hence, the experiments reported here extend the ones in Carraro and Bridge (2020), where we only report results for *ρ*^*p*^ = 0.5.

We also randomly split *D*^*mar*^ into three, i.e. *D*^*w*^, *D*^*val*^ and *D*^*gt*^ with proportions 15%-15%-70% respectively. Since the split is random, MAR distributions are preserved. *D*^*w*^ is used to calculate the weights for WTD (see Section 4.3 for details of the calculation). We use *D*^*val*^ as the validation set to optimize recommender system hyperparameters (Section 4.4). (In reality, the ratings one would use to optimize hyperparameters would either be a portion of *D*^*tr*^ or a portion of an intervened test set produced from *D*^*he*^. We decided it was better in the experiments that we report here to minimise the effect of hyperparameter selection on our results. Hence, we selected hyperparameter values using ‘unbiased’ data, *D*^*val*^).

We use *D*^*gt*^ as an unbiased test set. In other words, the performance of a given recommender on *D*^*gt*^ can be considered to be its “true”, unbiased performance (the ground-truth). We want the performance of a recommender on an intervened test set to be close to its performance on this unbiased test set. The best intervention strategy is the one that produces test sets where performance most closely resembles performance on *D*^*gt*^.

We train the five recommender systems presented in Section 4.4 using ratings in *D*^*tr*^. Each recommender produces a ranked list of recommendations which are tested on the unbiased test set *D*^*gt*^ and the intervened test sets. We have computed Precision, Recall, MAP and NDCG on the top-10 recommendations. Results are averaged over 10 runs with different random splits.

**Fig. 2:**
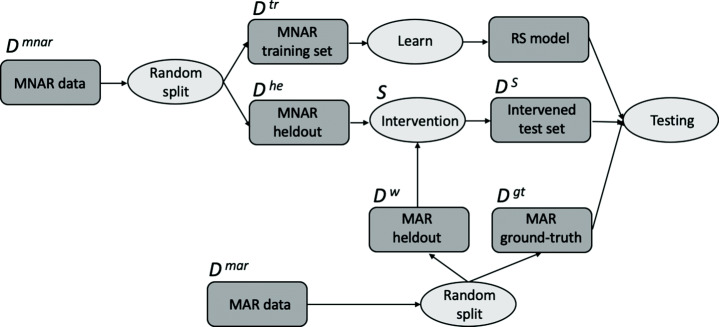
Visualization of the experimental methodology for each dataset

Figure 2 summarizes for each split the experimental methodology that we have just explained.

### Sampling strategies for the intervention

We formally present here the sampling strategies that we use to produce the intervened test sets in our experiments.^3^ Each strategy samples an intervened test set *D*^*S*^ from *D*^*he*^. For each strategy we give the corresponding probability sampling distribution, i.e. HCode $P_{S}({\mathscr{S}}|u,i)$. In addition to SKEW, WTD and WTD_H, we also employ two baselines. Regular (REG) is a random sample from *D*^*he*^, corresponding to an intervention that does not try to compensate for bias. FULL represents the test set in the classic evaluation, where the test set is *D*^*he*^ (therefore no intervention).

- **FULL**: $P_{S}({\mathscr{S}}|u,i) = 1$. A test set sampled with FULL is what one would use as a traditional test set. The computational time to calculate values for *P*_*S*_ is negligible.
- **REG:** $P_{S}({\mathscr{S}}|u,i) = 1 / |D^{he}|$. Every (*u*,*i*) has a constant probability of being sampled and so we obtain a test set that is a random subset of *D*^*he*^. We would expect this to behave very similarly to FULL test set, even though it is smaller. The computational time to calculate values for *P*_*S*_ is negligible.
- **SKEW:** $P_{S}({\mathscr{S}}|u,i) = 1 / |D^{tr}_{i}|$, where $|D^{tr}_{i}|$ counts the number of ratings that item *i* has in *D*^*tr*^ (Wang et al., 2018; Bonner & Vasile, 2018) (see also Section 2.5). The computational time to calculate values for *P*_*S*_ is $\mathcal {O}(M)$, with *M* being the number of items in the recommender scenario.
- **WTD**, **WTD_H**: $P_{S}({\mathscr{S}}|u,i) = w_{u} \times (w_{i})^{2}$. These are the two alternatives of our approach, presented in Sections 3.3.2 and 3.3.3. Weights are calculated using Formulas (13) and (14). WTD uses Formulas (1) and (2) to calculate the actual MAR posteriors from *D*^*w*^. WTD_H uses the hypothesized MAR posteriors instead. They both use Formulas (4) and (5) to calculate exact MNAR posteriors from *D*^*tr*^. The computational time to calculate values for *P*_*S*_ (i.e. the weights) is $\mathcal {O}(N + M)$, with *N* and *M* being the number of users and items in the recommender scenario, respectively.

Note that, in each of SKEW, WTD and WTD_H, if the distribution *P*_*S*_ does not sum to 1 (necessary for a probability distribution), we include a normalization step on *P*_*S*_ to ensure that this property is achieved.

### Recommender systems

We train five recommender models, all of them producing a ranked list of recommended items. AvgRating and PosPop are non-personalised recommenders which rank items in descending order of their mean rating and number of positive ratings in the training set, respectively. UB_KNN and IB_KNN are user-based and item-based nearest-neighbour algorithms (Cremonesi et al., 2010). MF is the Matrix Factorization algorithm proposed by (Pilászy et al., 2010). For UB_KNN, IB_KNN and MF we use the implementations available in the RankSys library.^4^ We used our own implementations of AvgRating and PosPop.

The UB_KNN, IB_KNN and MF algorithms have hyperparameters. We select hyperparameter values that maximize top-10 Recall on *D*^*val*^ (Section 4.2), using a grid-search tuning approach. For UB_KNN, IB_KNN, we choose the number of neighbors from {10,20,..,100}. For MF, we choose the number of latent factors from {20,40,..,200} and the regularization term from {0.001,0.006,0.01,0.06,0.1, 0.6}.

## Results

**Fig. 3:**
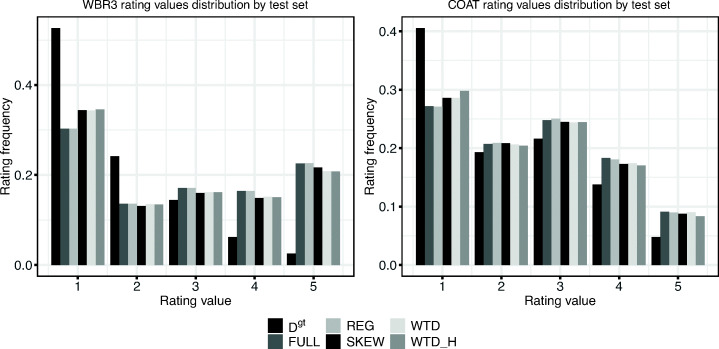
Distribution of rating values of the unbiased test set *D*^*gt*^, the baselines and the intervened test sets in WBR3 and COAT

**Fig. 4:**
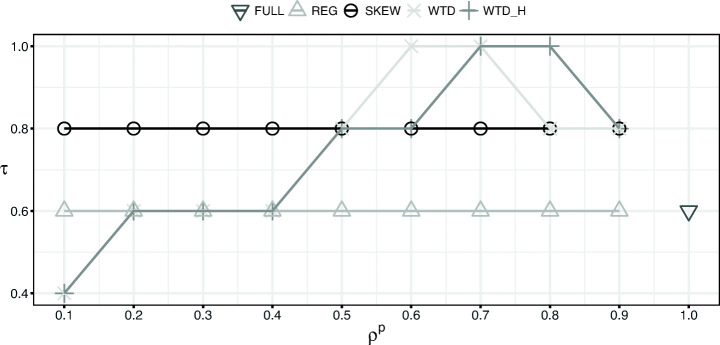
Kendall’s concordance coefficient (*τ*) values for WBR3

We report the results of our experiments in Figs. 3 and 4 and Tables 2 and 3.

**Table 2:** Kullback-Leibler (KL) divergence scores for WBR3 and COAT: scores represent the divergence of the baselines and the intervened test set rating values distribution with respect to the true unbiased rating values distribution of the unbiased test set *D*^*gt*^

	FULL	REG	SKEW	WTD	WTD_H
WBR3	0.417	0.417	0.359	0.347	0.343
COAT	0.072	0.073	0.058	0.06	0.047

**Table 3:** Recall@10 results for WBR3 and COAT

	*D*^*gt*^	FULL	REG	SKEW	WTD	WTD_H
WBR3						
PosPop	0.056	+ 280	+ 244(0.1)	+ 6(0.6)	-14(0.7)	+ 32(0.8)
AvgRating	0.016	-77	-76(0.8)	-5(0.1)	-1(0.2)	-3(0.2)
UB_KNN	0.073	+ 274	+ 239(0.1)	+ 5(0.3)	-15(0.5)	+ 7(0.6)
IB_KNN	0.071	+ 313	+ 270(0.1)	-1(0.2)	-8(0.5)	+ 11(0.6)
MF	0.077	+ 258	+ 226(0.1)	+ 23(0.1)	-8(0.3)	-15(0.4)
COAT						
PosPop	0.066	+ 133	+ 108(0.2)	+ 17(0.1)	+ 7(0.6)	-1(0.7)
AvgRating	0.068	+ 61	+ 44(0.1)	+ 15(0.2)	-6(0.1)	+ 9(0.2)
UB_KNN	0.067	+ 229	+ 210(0.1)	+ 117(0.3)	+ 9(0.3)	+ 1(0.3)
IB_KNN	0.073	+ 236	+ 208(0.3)	+ 99(0.2)	+ 4(0.2)	-1(0.3)
MF	0.063	+ 180	+ 154(0.3)	+ 122(0.3)	+ 60(0.1)	+ 49(0.1)

We report ground truth performances on test set *D*^*gt*^ in terms of Recall@10. We show the percentage difference of the best performances on the baselines and the intervened test sets with respect to *D*^*gt*^ In brackets, we give the test set proportion *ρ*^*p*^ where this best performance is achieved. For example, to explain two entries from the table, the PosPop recommender has a Recall@10 of 0.056 on the WBR3 ground truth, *D*^*gt*^; the Recall@10 of the same recommender on a test set that is produced by the WTD_H intervention method using *ρ*^*p*^ = 0.8 is 32% higher

To analyse the difference between the various sampling strategies, we plot the distribution of the rating values of each of the intervened test sets and we compare them with the unbiased test set *D*^*gt*^ (similarly to the analysis in Marlin et al. (2007)).

Firstly, Fig. 3 confirms the difference between unbiased (i.e. *D*^*gt*^) and biased distributions (i.e. FULL and REG) for both datasets. In general, unbiased distributions show a much higher proportion of low ratings than high ratings, confirming that in biased datasets users tend to rate items that they like (Marlin et al., 2007). This difference is less evident in COAT than WBR3 and we argue that this is due to the more artificial conditions under which COAT’s MNAR portion was collected (Schnabel et al., 2016) compared with the MNAR portion of WBR3. WBR3’s users experienced a standard recommender scenario (see Section 4.1) whereas COAT’s users were not influenced by a recommender. The COAT users, being Mechanical Turkers, are mere executors of a task and therefore less likely to care about their experience of using the system; therefore, we argue that COAT is more randomized and accordingly less biased (i.e. more similar to an unbiased dataset). To confirm those findings, we observe values for FULL and REG in Table 2 where we report Kullback-Leibler (KL) divergence scores between the intervened sets and the ground truth for both datasets. This KL divergence is much greater for WBR3 (approximately 0.4) than it is for COAT (approximately 0.07).

Compared with FULL and REG, the distributions of rating values in the intervened test sets (i.e. SKEW, WTD and WTD_H) are closer to the distribution in the unbiased ground truth for both datasets (although only to a limited extent): this can be observed in both Fig. 3 and Table 2. Such results show the first evidence that intervention might be a good solution to unbiased evaluation. Indeed, the results we present in detail later (i.e. Table 3 and Fig. 4) confirm that the relatively small increase in similarity between the SKEW, WTD and WTD_H test sets and the unbiased ground truth in terms of posteriors leads to a greater and much more appreciable similarity in the accuracy of the recommendations.

In Table 3, for each recommender, we show its ground-truth Recall@10 performance on the unbiased test set *D*^*gt*^ and its relative performance (in terms of percentage difference) on the baselines and intervened test sets with respect to this ground-truth. For each of REG, SKEW, WTD and WTD_H, we show the best performance among the ones obtained in the 10 different test sets (one for each different *ρ*^*p*^) and we show in brackets the test set size *ρ*^*p*^ for which this best performance is achieved. Results for Precision, NDCG and MAP are omitted because the percentage differences have a very similar trend to the Recall ones. The statistical significance of the results is assessed by performing a pairwise comparison test between the performance of each recommender on the five different test sets, i.e. the baseline sets (FULL, REG) and the intervened sets (SKEW, WTD and WTD_H). For such tests, we use a two-tailed Wilcoxon signed rank test^5^ with *p* < 0.05, and the results are reported in Table 4.

**Table 4:** The statistical significance of the results is assessed by performing a pairwise comparison test between the performance of each recommender on the five different test sets, i.e. the baselines sets FULL (‘F’), REG (‘R’) and the intervened sets SKEW (‘S’), WTD (‘WT’) and WTD_H (‘WH’)

	WBR3					COAT					
	F	R	S	WT	WH	F	R	S	WT	WH	
F	–	–	–	–	–	–	–	–	–	–	PosPop
R		–	–	–	–		–	–	–	–	
S	T	T	–	–	–	T	T	–	–	–	
WT	T	T	T	–	–	T	T		–	–	
WH	T	T	T	T	–	T	T			–	
F	–	–	–	–	–	–	–	–	–	–	AvgRating
R		–	–	–	–		–	–	–	–	
S	T	T	–	–	–	T		–	–	–	
WT	T	T		–	–	T			–	–	
WH	T	T	T		–	T				–	
F	–	–	–	–	–	–	–	–	–	–	UB_KNN
R	T	–	–	–	–		–	–	–	–	
S	T	T	–	–	–	T	T	–	–	–	
WT	T	T	T	–	–	T	T	T	–	–	
WH	T	T	T	T	–	T	T	T		–	
F	–	–	–	–	–	–	–	–	–	–	IB_KNN
R	T	–	–	–	–	T	–	–	–	–	
S	T	T	–	–	–	T	T	–	–	–	
WT	T	T	T	–	–	T	T	T	–	–	
WH	T	T	T	T	–	T	T	T		–	
F	–	–	–	–	–	–	–	–	–	–	MF
R	T	–	–	–	–		–	–	–	–	
S	T	T	–	–	–	T		–	–	–	
WT	T	T	T	–	–	T	T	T	–	–	
WH	T	T	T	T	–	T	T	T		–	

We write ‘T’ where a statistical significant difference in the performance is found

Results on WBR3 show that WTD and WTD_H outperform SKEW only for the MF recommender (where all differences are statistically significant). This is however a good result if we consider that WTD and WTD_H are best at debiasing the evaluation of one of the most successful and widely-used recommenders in the literature (Koren et al., 2009). SKEW is superior to WTD and WTD_H for the PosPop and IB_KNN recommenders (with statistically significant differences). For the UB_KNN recommender, WTD_H and SKEW are equally good (their performances are not statistically significantly different) and superior to WTD; for the AvgRating recommender, all three are equally good because performances are not statistically significantly different from each other. The superiority of SKEW for PosPop is somehow expected because SKEW is an intervention that is specific to popularity-bias; its superiority for UB_KNN can be explained by a similar reason, i.e. UB_KNN has also been proved to be a recomender with a popularity-bias (Cañamares and Castells, 2017).

We also observe that SKEW obtains its best performances on intervened sets that are smaller than the ones of WTD and WTD_H. However, this fact could raise questions about the reliability of SKEW’s results due to discarding the majority of the available test data.

Comparing only WTD and WTD_H performances, we find that in general WTD is better than WTD_H, with the only exception being for the AvgRating recommender (where their performances are not statistically significantly different) and UB_KNN (where WTD_H is better than WTD).

The results for COAT in the lower half of Table 3 show that WTD and WTD_H are equally good because performances are not statistically significantly different from each other. Also, they more closely approximate the ground truth for the personalised recommenders but not for the non-personalised recommenders. Indeed, their performances are not statistically significantly different to the one of SKEW for PosPop and the ones of REG and SKEW for AvgRating.

Finally, in both datasets, baselines FULL and REG are very far from the ground-truth, showing that ‘intelligent’ intervention strategies provide an effective debiasing technique in offline evaluations. Indeed, SKEW, WTD, WTD_H achieve statistically significantly different performances with respect to FULL and REG with the exception of SKEW for MF on COAT. In general, FULL and REG have similar results, regardless of the fact that the best performances of REG is generally achieved on a test set which is much smaller than FULL (except for the one of AvgRating in WBR3). This means that what matters is the strategy that performs the sampling, rather than the sampling itself.

Figure 4 reports an additional investigation on the results of Table 3. An offline evaluation typically ranks recommender algorithms from best to worst. This helps to narrow the number of different recommender algorithms that needs to be evaluated in costly user trials and online experiments. In our case then, it is important that performance estimates on intervened test sets, not only get close to the ground truth performance, but also rank different recommenders in the same way they would be ranked by performance estimates on the unbiased test set.

Before seeing whether the ranking of the recommenders on intervened sets corresponds to their ranking on the ground truth, we wanted to make sure that the ground truth ranking was reliable. Thus, we first computed statistical significance tests on the ground truth ranking. The statistical significance of the results is assessed by performing a pairwise comparison test between the performances of the recommenders on the unbiased test set *D*^*gt*^, again using the two-tailed Wilcoxon signed rank test described earlier. Results of these tests are reported in Table 5. We found that, for WBR3, recommender performances are statistically significantly different from each other, except for the pair UB_KNN & IB_KNN. Unfortunately, for COAT, no recommender performance is statistically significantly different from any other, except for the pair MF & IB_KNN. We argue that this is due to the small size of the COAT training set. This means that for COAT there is no point in comparing the rankings produced by the different intervened test sets, because all recommenders are roughly equivalent according to the ground truth test set.

**Table 5:** Statistical significance results for WBR3 and COAT

	PosPop	AvgRating	UB_KNN	IB_KNN	MF	
PosPop	–	–	–	–	–	WBR3
AvgRating	T	–	–	–	–	
UB_KNN	T	T	–	–	–	
IB_KNN	T	T		–	–	
MF	T	T	T	T	–	
PosPop	–	–	–	–	–	COAT
AvgRating		–	–	–	–	
UB_KNN				–	–	
IB_KNN				–	–	
MF				T	–	

We perform a pairwise comparison test between the performances of the recommenders on the unbiased test set *D*^*gt*^. We write ’T’ where a statistical significant difference in the performance is found

We use Kendall’s concordance coefficient (*τ*) to compare the ground truth recommender ranking obtained on the unbiased test set with the ones produced by the different interventions. For the reasons above, Fig. 4 reports the results for WBR3 only: for each of the intervention approaches we show concordance coefficients obtained in their 10 different intervened test sets. The figure shows that the ‘intelligent’ interventions are superior to FULL and REG, i.e. SKEW, WTD and WTD_H have values no smaller than the ones of REG (with the only exception of WTD & WTD_H when *ρ*^*p*^ = 0.1).

In more detail, FULL, REG and SKEW have constant *τ* values (0.6, 0.6 and 0.8, respectively), with SKEW being the best of the three. WTD and WTD_H have different values, depending on the size of their test sets. In general, both are superior to SKEW from *ρ*^*p*^ = 0.9 down to *ρ*^*p*^ = 0.6, achieving perfect correlation (*τ* = 1) when *ρ*^*p*^ = 0.8 (WTD_H), *ρ*^*p*^ = 0.7 (WTD & WTD_H) and *ρ*^*p*^ = 0.6 (WTD). SKEW, WTD and WTD_H have *τ* = 0.8 for *ρ*^*p*^ = 0.6, but SKEW is superior to all the other strategies from *ρ*^*p*^ = 0.1 up to *ρ*^*p*^ = 0.4 inclusive. We would argue that, in general, the results obtained by our debiasing strategies are more valuable than those of SKEW and REG because they are superior when sampling most of the data available for testing (except for when *ρ*^*p*^ = 0.9, where SKEW, WTD and WTD_H achieve the same correlation value). Indeed, *ρ*^*p*^ values smaller than 0.5 can result in intervened test sets that are too small to give reliable results. At the same time, *ρ*^*p*^ values greater than 0.8 can result in intervened test sets that are too similar to a biased test set to provide substantially different results with respect to a biased evaluation.

## Conclusions

In this paper, we presented WTD and WTD_H, our new sampling strategies that generate intervened test sets with MAR-like properties from MNAR data. These intervened test sets are more suitable for estimating how a recommender would perform on unbiased test data. One of the sampling strategies, WTD, requires that some MAR-like data be available since it approximates posterior probabilities calculated from that data. The other strategy, WTD_H, approximates the probabilities that we expect MAR data to exhibit.

### Findings

The paper assesses the effectiveness of these two strategies and it assesses, for the first time, the effectiveness of an existing intervention strategy from the literature, namely SKEW, which samples from MNAR data in inverse proportion to item popularity. With the use of (essentially) unbiased test sets as ground-truth, we showed these three sampling approaches to be successful in mitigating the biases found in a classical random test set. In general, we found SKEW to be particularly good at reducing the bias for well-known recommenders that themselves suffer from a popularity-bias (i.e. PosPop and both nearest-neighbour recommenders (Cremonesi et al., 2010)). Item popularity-bias is the kind of bias for which SKEW was designed. But our new strategies are the most robust (even if sometimes by a limited extent) on various key recommenders (MF on WBR3 and all the personalized recommenders on COAT) since they most closely approximate their unbiased ground-truth performances. The WTD strategy requires MAR data, which is rarely available, but we found that WTD_H, which uses a hypothesized MAR distribution, does work well, so MAR data is not necessary.

Our approach brings several benefits. First of all, it enjoys low overheads.

- Its design is simple and easy to implement and it does not require any learning phase for the weights, contrary to some unbiased estimators which might require expensive learning (e.g. Schnabel et al. (2016), where propensities are found via logistic regression).
- Moreover, intervention reduces the computational costs of testing a recommender because it generates smaller test sets.

Another advantage of our approach is that it has high generality.

- It works for both implicit and explicit datasets because it is independent of the interaction values (e.g. ratings) in the dataset.
- Despite the fact that WTD and WTD_H are close to SKEW on some recommenders, our way of calculating weights is a better motivated heuristic than the one of SKEW and, unlike SKEW, it is not tailored to item popularity-bias.
- Our approach can be extended to training a recommender, without any modification. Training a recommender on an intervened training set, instead of on a classical biased training set, might improve the recommender’s model and therefore boost prediction or top-*n* recommendation performances.
- Intervened data can be used to train *existing* recommender systems and to test recommender systems using *existing* metrics. Debiased training and testing hence become widely applicable without designing special models and special metrics. This feature is particularly desirable when benchmarking new recommender approaches with respect to existing ones.

### Limitations of our study and future works

Our work focuses on helping researchers to build reliable offline experiments. A future step to reinforce the validity of our debiasing strategies is to run more experiments with different datasets, for example datasets that are larger or ones that come from other recommendation domains. However, even so, it is well-known that online experiments, such as A/B tests and user trials, are essential to give authentic insights into what has been investigated offline. Therefore, our studies should be extended with online experiments. This is work for the future.

In Section 2.3 we described how to collect unbiased-like datasets by using the forced ratings approach. We also highlighted that those datasets are usually small and that this collection approach can only work in specific domains. Despite our work on debiasing data and other works in the literature too, we argue there is still the need for more unbiased data, allowing for more experiments on the testing (and, eventually, the training) of RSs. When evaluating an RS, bigger unbiased datasets would give a more grounded reference of unbiased performance. Alternatives to the forced ratings approach that are applicable across more domains and that generate bigger unbiased datasets might be investigated too. Additionally, similar approaches to collecting unbiased implicit datasets might also be useful.

Given the availability of a dataset collected by the forced ratings approach, there is still room for discussion to what extent this dataset can be considered an unbiased ground-truth for an RS evaluation. As we have emphasized in this paper (see Section 2.3 in particular), such a dataset might still carry some bias which might affect findings of studies like ours. There is bias, for example, when a user rates an item she already knows (Loepp et al., 2018) or when items are rated in sequence. Even if we were able to remove many (if not all) of the effects of confounders from a dataset collection process in a real-world scenario, the resulting unbiased dataset still might not display a uniform rating probability in practice. For these reasons, we believe further research on bias and its intrinsic mechanisms in an RS scenario need to be properly addressed in the future.

Finally, another aim for the future is to investigate other ways of calculating the weights for WTD. An alternative might be using techniques developed for causal inference, e.g. Cortes et al. (2008, 2010). Also, given the generality of our approach, it would be interesting to assess the effectiveness of WTD and WTD_H at debiasing implicit datasets, to complement the investigation performed in this paper.

## Footnotes

1 Available on request from https://webscope.sandbox.yahoo.com

2 Available from https://www.cs.cornell.edu/~schnabts/mnar/

3 The implementation of the sampling strategies is available at https://github.com/cdiego89phd/wtd-debiasing-RS-eval

4 https://github.com/RankSys

5 The version of the Wilcoxon test that we use includes zero-differences in the ranking process and assigns a zero rank to those differences. Also, it splits the zero ranks between positive and negative ones.
